# Dietary restriction, caloric value and the accumulation of hepatic fat

**DOI:** 10.1186/1476-511X-11-2

**Published:** 2012-01-05

**Authors:** Leandro P Moura, Gabriella A Figueredo, Natália O Bertolini, Marilia Ceccato, Jessica R Pereira, Amanda Christine S Sponton, Maria Alice R de Mello

**Affiliations:** 1 Department of Physical Education, Universidade Estadual Paulista (UNESP), Avenida 24ª n° 1515, P.O. Box 199, Bela Vista, Rio Claro, Zip code: 13506-900, SP, Brazil

**Keywords:** Non-isocaloric diets, Dietary restriction, Non-alcoholic fatty liver disease

## Abstract

**Background:**

Studies using laboratory animals under what are considered to be "standard" conditions normally offer unrestricted amounts of food to the animals, which can lead to metabolic disorders. Moreover, standard diets have different compositions.

**Aim:**

Therefore, the aim of the present study was to assess the effects of two non-isocaloric diets (commercial Purina^® ^and AIN-93M), which are considered standard diets, on the accumulation of fat in the liver of rats when offered ad libitum or in a restricted amount.

**Methods:**

Thus, 40 Wistar rats (90 days old) were separated into 4 groups according to the amount of food offered (ad libitum or dietary restriction) and the type of diet (commercial diet, 3,028.0 kcal/g or AIN-93M, 3,802.7 kcal/g): animals fed the commercial Purina^® ^diet ad libitum (AP), animals fed restricted amounts of the commercial Purina^® ^diet (RP), animals fed the AIN-93M diet ad libitum (AD), and animals fed restricted amounts of the AIN-93M diet (RD). Dietary restriction consisted of pair-feeding the RP and RD groups with 60% of the total food consumed by the corresponding ad libitum groups.

**Results:**

Because of its higher carbohydrate and calorie content, AIN-93M was found to accelerate weight gain, reduce glucose tolerance and peripheral insulin sensitivity, and increase the amount of fat in the liver when compared to the commercial diet. Conversely, a 40% dietary restriction assisted in weight loss without causing malnutrition, contributing to an improved glucose tolerance and higher levels of HDL cholesterol.

**Conclusion:**

Therefore, differences in the amount of carbohydrates and calories provided by the diet can lead to important metabolic disorders, such as impaired tolerance and accumulation of hepatic fat, and dietary restriction improves serum and tissue lipid profiles in laboratory animals.

## Background

Non-alcoholic fatty liver disease (NAFLD) is characterised by the presence of fat droplets in the liver [1,2]. Lipid levels equal to or greater than 5% of an organ's weight [1,3] characterises steatosis, which may range from simple fat accumulation without any evidence of inflammation to necroinflammatory manifestations, including steatohepatitis. Steatohepatitis, in turn, may progress to fibrosis in 50% of cases, to cirrhosis in 15% of cases, and to liver failure in 3% of cases [4-8].

Hepatic fat accumulation can be found in 57.5 to 75% of obesity cases [6] due to alterations such as an increase in fat consumption, a decrease in oxidation, and lipid secretion by the liver [9] or other mechanisms such as oxidative stress, the organism's inflammatory response, and insulin resistance (IR) [10,11]. Increased carbohydrate consumption may lead to IR [12], where peripheral tissues show an abnormal response to insulin that may lead to glucose intolerance [13] and, consequently, to NAFLD [14-17].

The key for reducing the disorders that stem from insulin resistance, glucose intolerance, and NAFLD is a change in lifestyle, including regular participation in physical activity and caloric restriction [18-21].

Given the limitations of research involving humans, animal models, in particular rodents, have become important research tools for several areas of science.

Two types of "standard diets", a commercial diet for rodents (Purina^®^) and a diet proposed by the American Institute of Nutrition (AIN), AIN-93 [22], are often used in physiological research studies that use rodents as experimental models. The problem with labelling these two diets as standard diets is the difference in their caloric values, which is approximately 800 kcal/g (Purina^®^: 3,028.0 kcal/kg and AIN-93M: 3,802.7 kcal/kg). This discrepancy is mainly due to differences in the amount of sugar present in each diet (Purina: 437 g/kg and AIN-93M: 700 g/kg). As previously noted, a high carbohydrate intake contributes to the development of metabolic disorders and may induce NAFLD. Therefore, the aim of the present study was to assess the effects of two non-isocaloric diets considered to be standard (commercial Purina^® ^and AIN-93M) for studies involving rodents as experimental models, offered *ad libitum *or in restricted amounts on the development of non-alcoholic fatty liver disease in rats.

## Results

**Figure 1:**
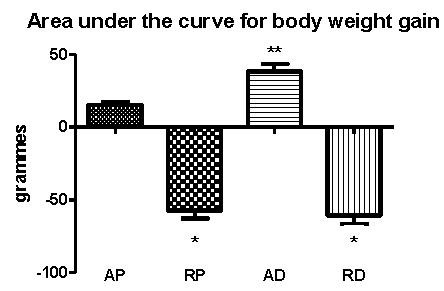
**Area under the curve for body weights from the whole experimental procedure**. AP = *ad libitum *pellet, RP = dietary restriction pellet, AD = *ad libitum diet, and *RD = dietary restriction diet. *≠ AP and AD and **≠ AP, RP, and RD (10 animals/group).

Animals fed *ad libitum *had higher weights than animals subjected to dietary restriction. When comparing both *ad libitum *groups, the group of animals fed the AIN-93M diet (AD group) was shown to have a larger weight gain (Figure 1).

**Figure 2:**
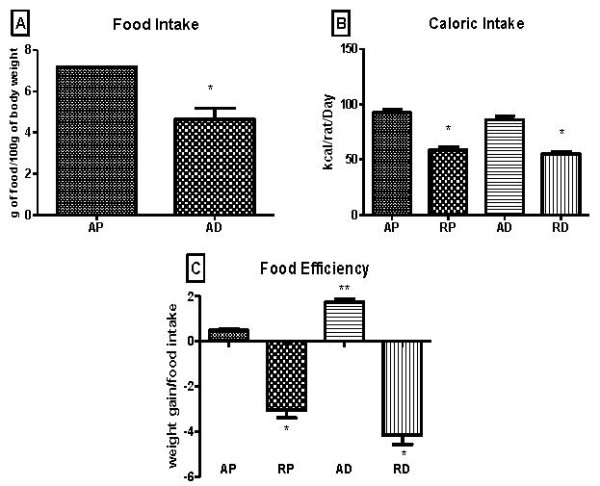
**Food intake (A), caloric intake (B), and food efficiency (C) during the whole experimental procedure**. AP = *ad libitum *pellet, RP = dietary restriction pellet, AD = *ad libitum *diet, and RD = dietary restriction diet. *≠ AP and AD and **≠ AP, RP, and RD (10 animals/group).

Food intake was lower in the AD group when compared to the AP group (Figure 2A). Regarding caloric intake, the amount of calories ingested by the groups subjected to dietary restriction was smaller than that of the *ad libitum *groups, as was expected (Figure 2B). The AIN-93M diet was shown to have a higher food efficiency than the commercial diet (Figure 2C).

**Figure 3:**
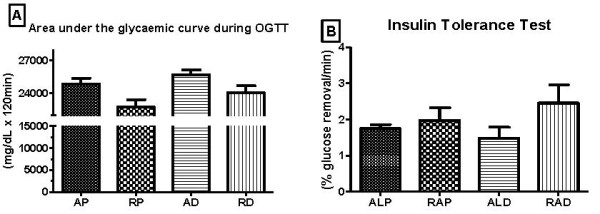
**Area under the glycaemic curve during glucose tolerance test (A) and % glucose removal during the insulin tolerance test (B) performed at the end of the experiment**. AP = *ad libitum *pellet, RP = dietary restriction pellet, AD = *ad libitum *diet, and RD = dietary restriction diet. *≠ AP, RP, and RD (10 animals/group).

Groups subjected to the dietary restriction presented a smaller area under the glucose curve during the oral glucose tolerance test (OGTT) (Figure 3A). However, when analysing the insulin tolerance test (ITT), no significant differences could be observed between the groups (Figure 3B).

**Figure 4:**
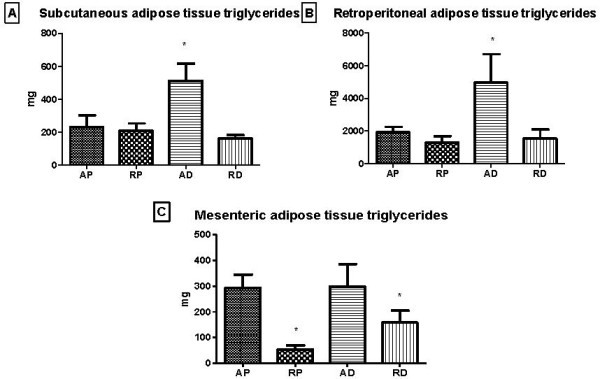
**Triglyceride concentrations of different adipose tissue depots, subcutaneous (A), retroperitoneal (B), and mesenteric (C), at the end of the experiment**. AP = *ad libitum *pellet, RP = dietary restriction pellet, AD = *ad libitum *diet, and RD = dietary restriction diet. *≠ AP and AD, ***≠ AP, RP, and RD (10 animals/group).

Regarding the amount of triglycerides contained in the subcutaneous (Figure 4A) and retroperitoneal (Figure 4B) adipose tissues, the AD group was found to have a higher triglyceride concentration when compared to the other groups. As for the mesenteric adipose tissue (Figure 4C), it was possible to observe a decrease in the triglyceride content in the groups subjected to dietary restriction.

**Figure 5:**
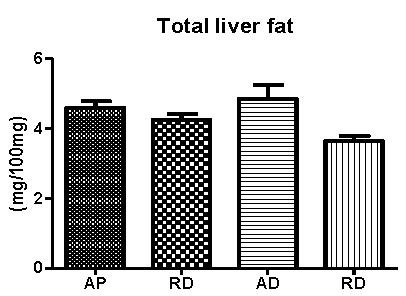
**Total hepatic fat concentrations at the end of the experiment**. AP = *ad libitum *pellet, RP = dietary restriction pellet, AD = *ad libitum *diet, and RD = dietary restriction diet. (10 animals/group).

The AD group presented higher concentrations of total hepatic fat when compared to the dietary restriction groups, and a tendency for an increase was noted when compared to the AP group (p = 0.067) (Figure 5).

Regarding the serum biochemistry, a greater concentration of HDL cholesterol could be observed in the groups subjected to the dietary restriction. No significant differences in LDL cholesterol, total cholesterol, triglycerides, glucose, total serum protein, and serum albumin levels could be observed among the studied groups (Table 1).

**Table 1:** Serum data obtained at the end of the experimental period

	AP	RP	AD	RD
**HDL (mg/dL)**	32.65 ± 3.07	38.35 ± 4.44*	31.50 ± 4.22	41.62 ± 3.91*
**LDL (mg/dL)**	36.43 ± 7.08	51.38 ± 8.11	42.96 ± 12.57	52.31 ± 7.76
**TG (mg/dL)**	101 ± 31.54	105.85 ± 27.02	96.64 ± 20.2	89.31 ± 28.75
**Total Cholesterol (mg/dL)**	65.56 ± 10.29	74.69 ± 11.79	62.44 ± 18.27	76.03 ± 11.28
**Glucose (mg/dL)**	82.97 ± 10.53	91.27 ± 23.21	90.17 ± 16.28	106.95 ± 15.86
**Total Protein (g/dL)**	6.79 ± 0.51	6.79 ± 0.16	6.85 ± 0.3	6.97 ± 0.26
**Albumin (g/dL)**	2.80 ± 0.39	2.75 ± 0.09	2.90 ± 0.15	2.91 ± 0.13

Data are expressed as mean ± standard deviation.

AP = *ad libitum *purina; RP = dietary restriction purina; AD = *ad libitum *diet, and RD = dietary restriction diet. *≠ AP and AD (10 animals/group).

## Discussion

Because there appears to be little concern with respect to the best way to feed animals used as "controls" in many scientific studies involving animal models, the present study aimed to assess the metabolic effects of dietary restriction in rats associated with the administration of two non-isocaloric diets (commercial Purina^® ^diet and semi-purified AIN-93M diet) that are considered standard diets for rodents. The main results of this study demonstrate that a 40% dietary restriction reduced weight gain without causing malnutrition, improved glucose tolerance, and increased the levels of circulating HDL cholesterol. The AIN-93M diet increased weight gain, reduced glucose tolerance, and led to the accumulation of fat in the liver when compared to the commercial diet due to its higher caloric and carbohydrate contents.

Serum albumin and total serum proteins were determined to assess the animals' nutritional statuses [23-25]. According to these results, animals subjected to the dietary restriction described in this study did not present malnutrition, given that no alterations in serum albumin and total serum proteins could be observed in any of the animals.

Regarding the serum lipid profile, increased levels of HDL cholesterol could be observed in the groups subjected to dietary restriction, demonstrating that a 40% dietary restriction does not cause malnutrition; this finding is in accordance with other studies [21-26], which also showed improvements in the lipid profiles of the animals.

In the present study, animals belonging to the *ad libitum *groups showed a larger weight gain when compared to animals in the groups subjected to dietary restriction. Animals fed the AIN-93M diet, which contains greater amounts of carbohydrates (26.3%) and calories (800 kcal/g of diet) [22] than the commercial diet, gained more weight when compared to those maintained with the commercial diet, when allowed to feed *ad libitum*. However, when subjected to dietary restriction, the effect of the diet on weight gain was inhibited.

The accumulation of body fat resulting from increased carbohydrate intake occurs due to the fact that excess carbohydrates, when not used by the organism, are initially degraded into pyruvate and then attached to coenzyme A (CoA) to become acetyl-CoA. Acetyl-CoA is transformed into malonyl-CoA with the help of the enzyme acetyl-CoA carboxylase. The fatty acid synthase enzyme then converts malonyl-CoA into palmitate. Following the esterification of palmitate with the α-glycerol phosphate in adipose tissue, triacylglycerol is formed and stored as an energy reserve [28].

The effects of both diets on the accumulation of body fat are more pronounced when analysing the total triglyceride concentrations of the three main fat depots in the organism (mesenteric, subcutaneous, and retroperitoneal). The effect of the high-calorie diet, AIN-93M, on stimulating the accumulation of fat when compared to the commercial diet became evident, as did the effect of dietary restriction in modulating such an accumulation.

Regarding the analysis of food consumption in the *ad libitum *groups, the AD group ingested smaller amounts of food than did the AP group. These data are in accordance with other studies [29], where rats fed high-calorie diets presented a lower food intake. However, no significant difference regarding caloric intake could be observed among the studied groups.

When considering food efficiency, meaning the animal's ability to transform ingested calories into body weight [30,31], animals belonging to the groups subjected to dietary restriction showed negative values, which were considered beneficial given that the aim of dietary restriction was to minimise weight gain. When comparing *ad libitum *groups, the animals that were fed the AIN-93M diet (AD) were more efficient in transforming ingested energy into body mass.

It is known that the accumulation of adipose tissue can lead to insulin resistance, given that the adipose tissue is an endocrine organ that secretes several pro-inflammatory substances that may interfere in the hormone's signalling pathway and lead to a resistance to its actions [32-34]. The present study showed that the greater weight gain observed in the *ad libitum *groups compared to the groups subjected to dietary restriction was not sufficient to cause insulin resistance. This may be because these rats were not obese and showed a normal weight gain for their age [35]. Conversely, when assessing glucose tolerance, impairments deriving from the administration of the AIN-93M diet were identified.

The OGTT showed that rats fed the AIN-93M diet *ad libitum *had a lower glucose tolerance when compared to those fed the commercial diet. According to [36,17], the chronic administration of a carbohydrate-rich diet may lead to glucose intolerance. Given this substrate's high availability, if it is not used by the organism, its transformation into fat may cause an increase in the availability of circulating free fatty acids and an increase in adipose tissue, as was observed in the present study. This may lead to a malfunction of the insulin-signalling pathway [37]. A tendency for a reduction in peripheral sensitivity to the hormone could be observed in the AD group (p = 0.064) when compared to the AP group. If the experimental period had been extended, insulin resistance may have been observed in this group, which would explain the glucose intolerance.

Non-alcoholic fatty liver disease (NAFLD) is characterised by the accumulation of fat in the liver, which may lead to inflammation, fibrosis, and organ failure [4-8]. There is no specific treatment for this disease to date. Therefore, it is clear that research involving laboratory animals is of great importance to increasing knowledge about this disease [38].

It is known that hepatic lipogenesis is activated following the consumption of carbohydrates because the resulting increase in insulin secretion activates the lipogenic transcription factor, SREBP-1c [39,40]. In individuals with insulin resistance, the situation is aggravated by increased circulating insulin levels, which stimulate the synthesis of triglycerides and increases the secretion of VLDL cholesterol, contributing to a greater accumulation of fat in the liver [12].

No significant differences in the accumulation of hepatic fat were observed in the present study, though a strong statistic tendency could be noted (p = 0.067). If the experiment was extended, it may have shown that the AIN-93M diet would most likely lead to insulin resistance and, consequently, a greater accumulation of fat in the liver due to its high carbohydrate content when compared to the commercial Purina^® ^diet [12].

## Conclusion

The present study illustrates that differences in the amounts of carbohydrates and calories provided in a diet may lead to important metabolic changes, such as impaired tolerance and accumulation of hepatic fat, and that dietary restriction improves serum and tissue lipid profiles in laboratory animals.

## Materials and method

### Animal treatment

Forty Wistar rats, which were 90 days old at the beginning of the study, were housed in polyethylene cages measuring 37 × 31 × 16 cm (five rats per cage), at room temperature (25°C) with a 12-hour light/dark photoperiod. All procedures performed on the animals were submitted to and approved by the Animal Research Ethics Committee (CEUA) of the Institute of Biosciences at UNESP, Rio Claro campus (Process number: 2011/6274).

### Experimental design and groups

The animals were separated, according to the dietary protocol, into four groups (10 per group): animals fed the commercial Purina^® ^diet *ad libitum *(AP), animals fed restricted amounts of the commercial Purina^® ^diet (RP), animals fed the AIN-93M diet *ad libitum *(AD), and animals fed restricted amounts of the AIN-93M diet (RD).

The daily food consumption of the AP and AD animals was recorded. The following day, animals in the dietary restriction groups were pair-fed an amount equivalent to 60% of the previous day's average intake of the respective *ad libitum *group. This protocol was chosen because it provides dietary restriction without causing malnutrition and increases the animal's quality of life [41-45]. All animals were offered free access to water, regardless of their group.

### Diet Compositions

*Commercial Purina^® ^Diet (Paulínia/SP, Brazil): *This diet was composed of 43.7% carbohydrates, 23% protein, and 4% fat at 3,028 kcal/g. The remainder of the ingredients were comprised of minerals, fibre, and vitamins.

*AIN-93M (Semi-purified diet, according to the American Institute of Nutrition, AIN-93M *[22]): The diet was composed of 70% carbohydrates, 14% protein, and 4% fat at 3,802.7 kcal/g. The remainder of the ingredients were comprised of minerals, fibre, and vitamins.

### Oral Glucose Tolerance Test - OGTT

An oral glucose tolerance test (OGTT) was performed on the animals at the end of the experiment after a 12-hour fast. An initial blood sample was obtained through a small cut at the end of the animal's tail. A glucose solution (80%) was then administered to each animal in the amount of 2 g/kg of body weight using a polyethylene gastric tube. Blood samples were collected 30, 60, and 120 minutes later using heparinised capillaries calibrated to 25 μL for determining glucose concentrations. A single cut at the end of the tail was sufficient for all sample collections. Blood glucose concentrations were determined by the glucose oxidase method using commercial kits (Laborlab^®^). Results were analysed by calculating the area under the glucose curve using the trapezoidal method [46] with the help of the Origin 6.0 software (2000).

### Insulin Tolerance Test - ITT

Insulin sensitivity was evaluated through the insulin tolerance test (ITT). The ITT was performed at the end of the experiment and 48 hours after the OGTT. An initial blood sample was obtained by making a cut at the end of the animal's tail (time 0). An insulin solution, at the dose of 150 mU/100 g body weight, was then administered through an intraperitoneal injection. New blood samples were obtained after 4, 8, 12, 16, and 20 minutes using heparinised capillaries calibrated to 25 μL for determining the glucose concentrations by using commercial kits (Laborlab^®^). A single cut at the end of the tail was sufficient for all sample collections. The results obtained were analysed by calculating the glucose removal rate (KITT). KITT was expressed as %/minute and was calculated using the formula (0.0693/t_1/2_) × 100. Glucose removal (t_1/2_) was calculated by a least squares analysis of the curve of the blood glucose levels during the period of insulin decay following its administration [47], using the Origin 6.0 software.

### Evaluation of the nutritional state

Body mass and food intake were recorded daily throughout the experimental period. The results were analysed by calculating the areas under the curves for body mass and food intake throughout the experiment using the trapezoidal method [48]. Food efficiency was calculated to evaluate the animal's ability to convert consumed food energy into body weight. This calculation was made according to the World Health Organization [30], by dividing the average weekly weight gain of animals within each box (g) by the total energy intake (kcal). The energy intake was calculated by multiplying the amount of food ingested by the caloric value of each diet.

At the end of the experiment, all animals were anaesthetised with CO_2 _prior to being euthanised by decapitation. Blood samples were collected for the determination of blood glucose, triglycerides, total cholesterol, LDL cholesterol and HDL cholesterol levels by using commercial kits (Laborlab^®^, São Paulo/Brazil) and for total serum protein and serum albumin measurements. Samples for determining total lipids were also collected from the liver and from different depots of adipose tissue (mesentery, subcutaneous, and retroperitoneal) for triglyceride analysis [49].

### Statistics

The normality of the data was confirmed by the Shapiro-Wilk test. The results are presented as mean ± standard deviation. Comparisons between groups were made through an analysis of variance (one-way ANOVA) and the Newman-Keuls post-hoc test when necessary. A predetermined 5% significance level was used for all the analyses. The statistical programme used was the SPSS 17.0.

## List of abbreviations

NAFLD: *nonalcoholic fatty liver disease*; IR: *insulin resistance*; AIN: *American Institution of Nutrition*; oGTT: *oral glucose tolerance test*; ITT: *insulin tolerance test*.

## Competing interests

The authors declare that there are no conflicts of interest regarding the present study.

## Authors' contributions

All of the authors contributed to the study, not only with regard to sample collections but also with regard to the preparation of this manuscript. All of the authors have read and approved of the final version of this manuscript.
